# Comparison of Outcomes Between Anti-Nuss Operation and Modified Anti-Nuss Operation Using a Flexible Plate for Correcting Pectus Carinatum: A Retrospective Study

**DOI:** 10.3389/fsurg.2020.600755

**Published:** 2021-02-15

**Authors:** Lei Wang, Juan Liu, Saie Shen, Yao Li, Tienan Feng, Guoqing Li, Haibo Xiao, Fengqing Hu

**Affiliations:** ^1^Department of Cardiothoracic Surgery, Xinhua Hospital Affiliated to Shanghai Jiao Tong University School of Medicine, Shanghai, China; ^2^Department of Nursing, Shanghai Baoshan Hospital of Integrated Traditional Chinese and Western Medicine, Shanghai, China; ^3^Department of Anesthesiology, Xin Hua Hospital Affiliated to Shanghai Jiao Tong University School of Medicine, Shanghai, China; ^4^Department of Disaster and Emergency Medicine, Shanghai East Hospital, Tongji University, Shanghai, China; ^5^Clinical Research Institute, Shanghai Jiao Tong University School of Medicine, Shanghai, China; ^6^Second Affiliated Hospital of Chengdu Medical College, China National Nuclear Corporation 416 Hospital, Chengdu, China

**Keywords:** anti-Nuss operation, modified anti-Nuss operation, pectus carinatum, surgical correction, minimally invasive

## Abstract

**Introduction:** The anti-Nuss procedure has gradually been found to have several shortcomings in clinical practice. Accordingly, our department previously designed and introduced a new steel plate. However, there is limited evidence regarding its safety and efficacy. Thus, we aim to compare the efficacy and safety of the conventional anti-Nuss operation with those of a modified anti-Nuss operation using a flexible plate.

**Methods:** Patients with pectus carinatum who underwent surgery between January 2014 and August 2019 were consecutively enrolled in this single-center, retrospective study. In all, 53 patients underwent the modified procedure using the new steel plate (new procedure group), whereas 43 underwent the conventional anti-Nuss procedure (traditional procedure group). Outcome analysis was performed using SPSS to compare the intraoperative and postoperative short-term outcomes.

**Results:** All patients in the new procedure group had shorter operation duration (75.23 ± 11.90 vs. 82.45 ± 9.30 min, *p* = 0.008), postoperative hospitalizations (3.42 ± 0.95 vs. 4.64 ± 1.53 days, *p* = 0.039), and plate removal surgery durations (40.60 ± 3.47 vs. 60.30 ± 9.75 min, *p* = 0.041) than patients in the traditional procedure group. There were no significant differences in the length of incision, postoperative Haller index, cost, postoperative surgical outcome, and incidence of complications between the two groups.

**Conclusion:** Our data reveal that the main clinical outcomes were similar for after anti-Nuss operation and modified anti-Nuss operation. However, the modified procedure for pectus carinatum had a shorter operation duration, postoperative hospitalization, and plate removal surgery duration.

## Introduction

Nowadays the anti-Nuss operation has been widely used as standard surgery for pectus carinatum because of its minimally invasive nature (1, 2). However, shortcomings of this procedure have gradually been found in clinical practice (3, 4). For instance, the traditional steel plate without the curved portion is required to be shaped with special tools before the operation; this increases the duration of operation. During the operation, it is hard to push the steel plate through the anterior sternum, and it is hard to fix the steel plate firmly just by using a steel wire. Moreover, after the operation, the traditional rigid steel plate often limits the growth and development of the chest wall in children. Besides, it takes a long period for surgeons to place or extract the steel plate (5, 6).

To address these above mentioned shortcomings, we designed a new steel plate and introduced a corresponding modified surgery procedure. The new steel plate is easy to be shaped before or during the procedure; this plate is flexible causing less limitation to the growth and development of the children's thorax. Furthermore, the plate can be fixed more firmly by screws as well as steel wires and fixing pieces. Accordingly, in this study, we aimed to compare the safety and efficacy of this modified procedure with the new steel plate with those of the conventional anti-Nuss operation.

## Patients and Methods

### Patient Groups

This is a single-center clinical cohort study based on the retrospective analysis of prospectively collected data on patients with pectus carinatum who underwent surgical correction between January 2014 and August 2019. A total 96 patients were identified from databases. Of these, 53 patients underwent the modified anti-Nuss procedure and were included in the new procedure group, whereas 43 patients underwent the traditional anti-Nuss procedure and were included in the traditional procedure group. The operation type selected by the patient was based on will of the family and surgical indication. The chest wall plasticity test was performed by two experienced surgeons before operation, and the shape of chest after pressing the sternum into chest wall was the same as that after operation. Preoperative examination of patients included blood routine analysis, electrolyte analysis, liver and kidney function tests, heart color ultrasound, chest CT, and electrocardiography. Pulmonary function examinations were not performed as young patients were not suitable for these. The patients included in this study were aged between 10 and 18 years as patients under the age of 10 years have a larger space for sternal development and a greater risk of recurrence after plate removal. Pectus carinatum patients with pectus excavatum, those with recurrent pectus carinatum, and those with a history of chest and sternum surgery were excluded. All patients were followed up for 3 months after the operation to observe the wound healing and the displacement of the steel plate via chest CT. Subsequently, chest CTs were performed every 6 months until the removal of the steel plate. The study protocol was approved by the Institutional Review Board of Xinhua Hospital (Approval No. XHEC-D-2020-106), Shanghai Jiao Tong University School of Medicine, and informed consent was obtained from all patients.

### New Steel Plate Configuration and Accessories

**Figure 1:**
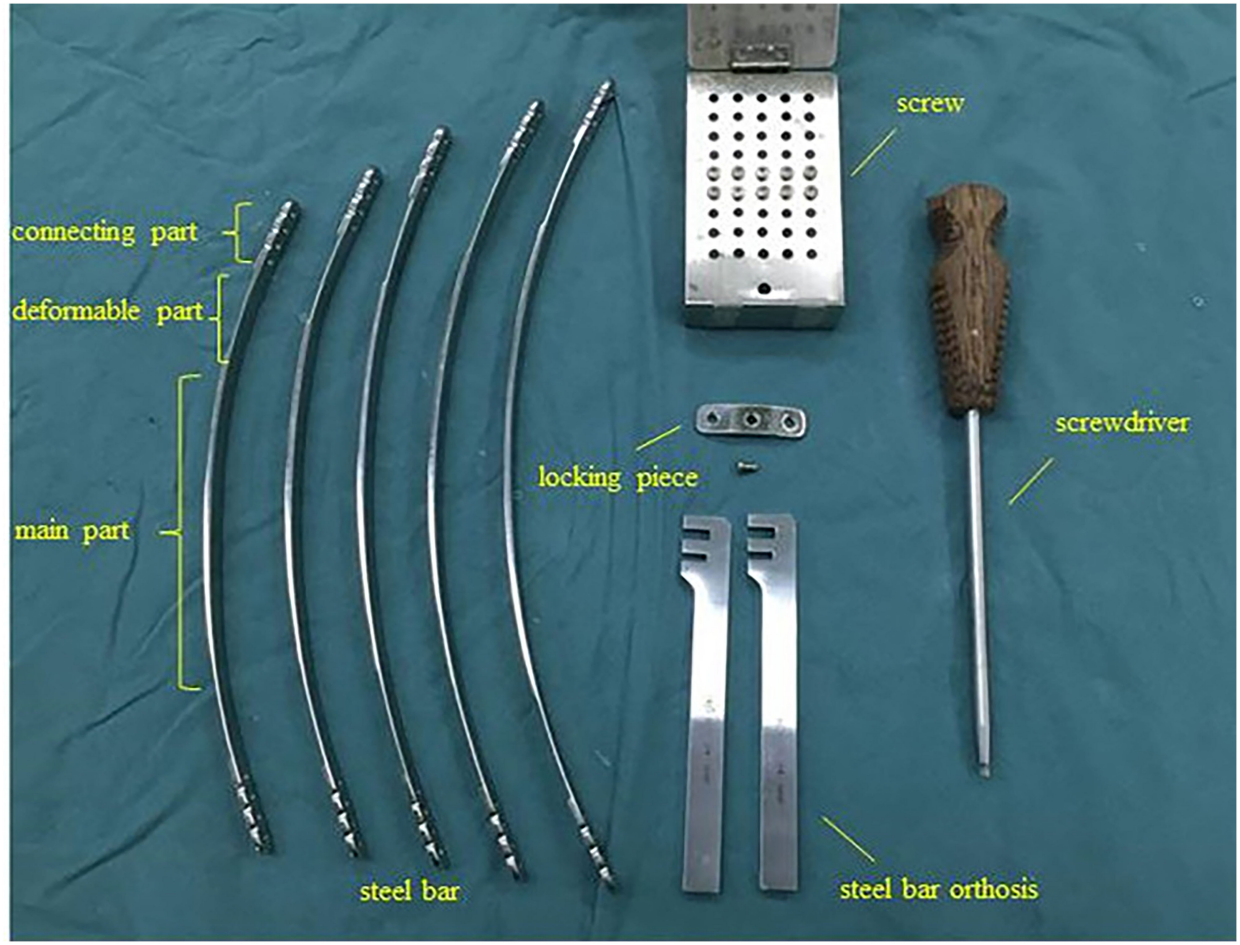
Bar configuration and accessories.

The new steel plate comprised three parts: main part, deform-able part and connecting part. Main part of the new steel plate is a curved steel plate with an arc similar to the physiological curvature of the front chest wall of a healthy person. The deformable part is an extension of the main part at the two terminals, which is thin and soft, and can be curved into a desired shape with hands or using simple tools. The deformable part extends outward to the connecting portion of the new steel plate and the thickness of the connecting part is the same as that of the main part. The connecting part contains a longitudinal arrangement of four screw holes, which can be used to fix the steel plate with the help of screws and a locking piece. Furthermore, the degree of settlement of the chest wall can be adjusted through different screw holes. The steel bars are available in the lengths of 28, 30, 32, 34, and 36 cm. The related accessories are as follows: the fixing piece is straight with a groove in the middle and can be connected to the connection part using screws. Steel plate orthosis contains two card slots which can be used for the orthopedics of steel plates. Screws and screwdrivers for the corresponding locking piece and side hole of the steel bar are also included (Figure 1).

### Modified Surgical Procedure

**Figure 2:**
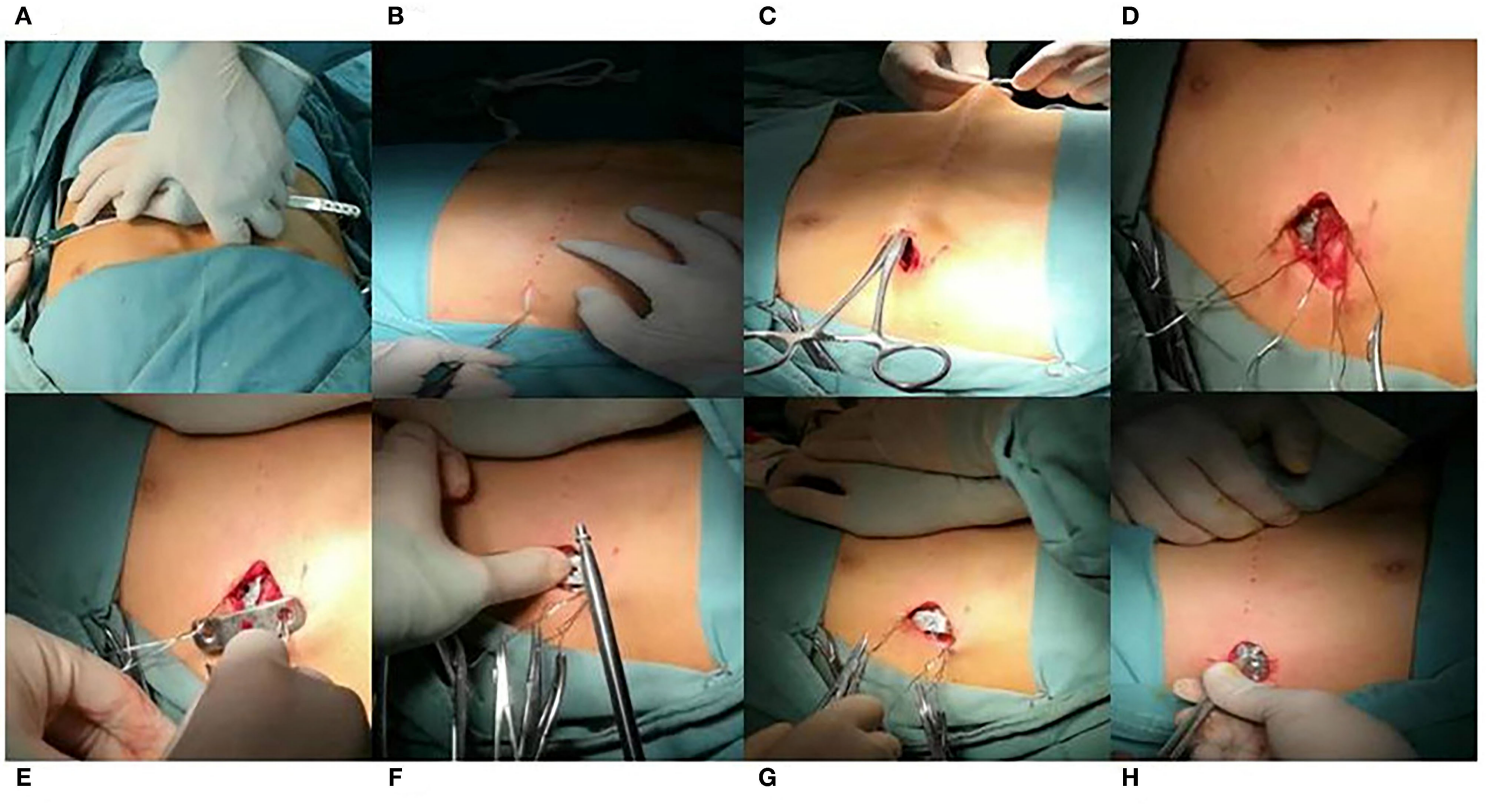
Modified procedure for pectus carinatum. **(A)** Press the highest point of the sternum to make the profile of the chest wall as normal so as to measure the length between two sides of the incision with a long rope, whose length was the same as the steel plate we used in the operation. **(B)** The vertical incision along the midaxillary line was adopted. **(C)** Build a subcutaneous tunnel with the help of a oval forceps tied by a long rope which is reserved through the subcutaneous tunnel. **(D)** Upper and lower ribs of each surgical incision are sutured with two steel wires. **(E)** One of the steel wires of each side is placed in the hole of the locking piece for later use. **(F)** Place the fixing screw after aligning the fixing piece against the joint of steel bar with the screw hole. **(G)** The fixing piece was also fastened to the adjacent two ribs with steel wires. **(H)** If the thorax shape is not satisfactory, the degree of sedimentation of the chest wall could be adjusted through the lateral hole of the plate to achieve the best corrected shape.

The patient was placed in supine position and the upper limbs were abducted at 90° after combined general anesthesia and tracheal intubation. We marked the highest point of the thoracic and the surgical incision on both sides. Generally, a vertical incision along the midaxillary line was adopted and the length of the incision was ~3 cm. Press the highest point of the sternum to make the profile of the chest wall as normal (Figure 2A). Meanwhile, we measured the length between the two sides of the incision using a long rope, and a steel plate of the same length was used in the operation. Following this, surgical incisions were made on both sides along the marked line and the subcutaneous tissue was separated to explore the surface of the muscle (Figure 2B). We then built a subcutaneous tunnel with the help of oval forceps tied with a long rope, which was reserved through the subcutaneous tunnel (Figure 2C). The upper and lower ribs of each surgical incision were sutured with two steel wires (four wires on each side) (Figure 2D). Further, one of the steel wires on each side was placed in the hole of the locking piece for later use (Figure 2E). The selected steel plate was then introduced with the help of the rope through the subcutaneous tunnel and the rope was then removed. A surgical staff member pressed the highest point of the sternum again, bringing the chest wall to the ideal state. At the same time, the other operator shaped the steel plate appropriately and placed the fixing screw after aligning the fixing piece against the joint of the steel plate with the screw hole on one side of the chest wall (Figure 2F). The screw was passed through the central hole of locking piece, and then into one of the holes in the steel bar. It is noteworthy that the screw was placed next to the rib but not lodged in the rib (Supplementary Figure 1 and Supplementary Video). The fixing piece was also fastened to the adjacent the two ribs with steel wires (Figure 2G). The same method was performed to fix the contralateral locking piece to complete the pectus carinatum orthosis. If the shape of the thorax was not satisfactory, the degree of sedimentation of the chest wall could be adjusted through the lateral hole of the plate to achieve the best corrected shape (Figure 2H). Finally, the incision was sutured and the operation completed.

### Statistical Analysis

Epidata 3.1 was used for data entry; and SPSS 20.0 statistical software was used for data analysis. Basic information included sex, age, type of operation, and preoperative Haller index. We evaluated the outcomes as follows: protuberance of the sternum on Chest CT, symmetry of the morphology of the chest wall, depression status, satisfaction of patients and their families, and the extension and elasticity of thorax appearance. The outcomes were considered excellent, good, fair, or poor if 4, 3, 2, or 1/0 criteria were positive, respectively. The continuous variables with normal distribution are expressed by mean ± standard deviation. The continuous variables with non-normal distribution are expressed by medians and interquartile ranges (IQRs). The count data are expressed as number of cases (*n*) and percentage (%). The comparisons among normally distributed continuous variables were conducted via *t*-test or ANOVA, whereas those among non-normally distributed variables were conducted via Mann–Whitney *U* test or Kruskal-Wallis test. Comparisons between enumeration data were conducted by Chi-Square or Fisher exact method. Finally, *p* < 0.05 was considered to be statistically significant.

## Results

### Study Characteristics

All 96 patients were successfully treated. In the new procedure group, there were 50 males and three females (average age, 13.83 ± 1.57 years; range, 10–17 years). The type of symmetry (*n* = 48) accounted for 90.57% of all patients (*n* = 53), and the preoperative Haller index was 2.07 ± 0.21. In the traditional procedure group, there were 41 males and two females (average age, 13.90 ± 1.75 years; range, 11–18 years). The type of symmetry (*n* = 39) accounted for 90.70% of all patients (*n* = 43), and the preoperative Haller index was 1.90 ± 0.31. There were no significant difference between groups in terms of age, sex, symmetry of chest wall, and the preoperative Haller index (*p* > 0.05) (Table 1).

**Table 1:** Comparison of patient information between new procedure and traditional procedure group.

	**New procedure group (*n* = 53)**	**Traditional procedure group (*n* = 43)**	***p*-value**
Sex			
Male	50 (94.34)	41 (95.35)	
Female	3 (5.66)	2 (4.65)	1.000
Age (years)	13.83 ± 1.57	13.90 ± 1.75	0.218
Type			
Symmetry	48 (90.57)	39 (90.70)	1.000
Asymmetry	5 (9.43)	4 (9.30)	
Preoperative Haller index	2.07 ± 0.21	1.90 ± 0.31	0.312

### Intraoperative and Short-Term Outcome Comparison

**Figure 3:**
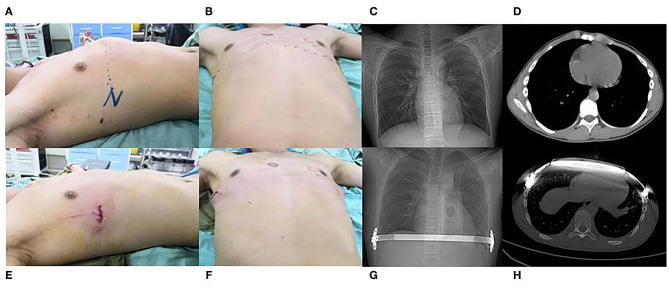
Appearance **(A,B)** and chest scan **(C,D)** of a 14-year-old pectus carinatum patient before modified anti-Nuss procedure; Appearance **(E,F)** and chest scan **(G,H)** of the 14-year-old pectus carinatum patient after modified anti-Nuss procedure.

In the new procedure group, the operation duration was 75.23 ± 11.90 min, the length of incision was 3.37 ± 0.38 cm, the postoperative Haller index was 2.78 ± 0.33, the postoperative hospitalization duration was 3.42 ± 0.95 days, the cost was 45,660.00 ± 1,855.00 yuan, and plate removal surgery duration was 40.60 ± 3.47 min. The postoperative surgical outcomes were good in 45 patients (84.91%) and fair in eight patients (15.09%) in this group (Figure 3). Further, in the traditional procedure group, the operation duration was 82.45 ± 9.30 min, length of incision was 3.40 ± 0.44 cm, postoperative Haller index was 2.52 ± 0.32, postoperative hospitalization duration was 4.64 ± 1.53 days, cost was 45,983.00 ± 1,379.00 yuan, and plate removal surgery duration was 60.30 ± 9.75 min. The postoperative surgical outcomes were good in 36 patients (83.72%) and fair in seven patients (16.28%). We found no significant difference in the length of incision, postoperative Haller index, cost, and postoperative surgical outcomes (event of displacement of the steel plate) between the two groups (*p* > 0.05) (Table 2). However, the new procedure group showed a shorter operation duration, postoperative hospitalization duration, and plate removal surgery duration compared with the traditional procedure group, with acceptable postoperative surgical outcomes (Table 2) (*p* < 0.05).

**Table 2:** Comparison of surgical characteristics between new procedure and traditional procedure group.

	**New procedure group (*n* = 53)**	**Traditional procedure group (*n* = 43)**	***p*-value**
Operation time (min)	75.23 ± 11.90	82.45 ± 9.30	0.008
Length of incision (cm)	3.37 ± 0.38	3.40 ± 0.44	0.667
Postoperative Haller index	2.78 ± 0.33	2.52 ± 0.32	0.673
Postoperative hospital stay (day)	3.42 ± 0.95	4.64 ± 1.53	0.039
Cost (yuan)	45,660.00 ± 1,855.00	45,983.00 ± 1,379.00	0.659
Operation time for plate removal (min)	40.60 ± 3.47	60.30 ± 9.75	0.041
Event of the displacement of the steel plate			1.000
Yes	0	0	
No	53	43	
Postoperative surgical outcome			1.000
Good	45 (84.91)	36 (83.72)	
Fair	8 (15.09)	7 (16.28)	
Poor	0	0	

### Complication Comparison

No significant difference was observed between groups in the incidence of complications (*p* > 0.05). In the new procedure group, two cases of postoperative wound infection, one case of bar displacement, and one case of bar exposure caused by delayed wound healing were observed. No intervention was required in cases with pneumothorax. Debridement was performed in the two cases with wound infection and the case with bar exposure. The patient with obvious bar displacement required reoperation and repositioning of the bar outside the proximal rib with a wire. All other patients recovered smoothly. In the traditional procedure group, one case of pneumothorax required treatment and three cases of wound infection or bar exposure caused by delayed wound healing were observed. All cases of wound infection or bar exposure received debridement, and the case with bar displacement was treated via reoperation and repositioning of the bar (Table 3).

**Table 3:** Complications in new procedure and traditional procedure group.

	**New procedure group (*n* = 53)**	**Traditional procedure group (*n* = 43)**	***p*-value**
Complications			0.508
No	49 (92.45)	38 (88.37)	
Yes	4 (7.55)	5 (11.63)	
Complications			
Pneumothorax	0 (0.00)	1 (20.00)	
Pleural effusion	0 (0.00)	0 (0.00)	
Wound infection	2 (50.00)	2 (40.00)	
Bar displacement	1 (25.00)	1 (20.00)	
Bar exposure due to delayed Wound healing	1 (25.00)	1 (20.00)	

## Discussion

For a long time, the Ravitch procedure or its modified versions have been considered as classic correction procedures for treating pectus carinatum. The main steps included in this procedure are the elevation of pectoralis major muscles, subperichondrial resection of defective costal cartilages, and transverse sternal osteotomies. This procedure, however, has the disadvantages of a long operating duration, long hospitalization, high blood loss, and scarring of the anterior chest wall. In the last 10 years, minimally invasive correction of pectus carinatum has gained popularity. The traditional anti-Nuss procedure using the Nuss plate has since been used for sternal deposition (7–9). However, this procedure and the plate have also been reported to have some shortcomings (10, 11). For example, the steel plate without the curvature needs to be shaped using special tools before the operation, and it is usually hard to push the steel plate through the anterior sternum (12, 13). Besides, it is often difficult for surgeons to fix the steel plate firmly by just using steel wires and it usually takes a long time to place or withdraw the plate (14, 15). Keeping in mind these shortcomings, we have previously designed an improved steel plate and modified the traditional procedure to achieve better results.

The new steel plate was key for the modified anti-Nuss procedure. The curved portion, deformable portion, and the screw fixation were important for the new steel bar. First, the traditional steel plate needed to be shaped before operation and because of the hardness of the integral steel plate, it required a strong force to be shaped. However, we found that the junction between the lateral chest wall and the front chest wall may be the key part and possibly play the most important role in the shaping of thorax. As a result, we designed and invented the curved and deformable portions of the steel plate. For curved portion of the steel plate, we designed the arc to be similar to the physiological curvature of the front chest wall of a healthy person. The deformable portion was the extension of the main part at the two ends, which was thin and soft, and could be curved into a desired shape using simple tools both before or during the operation. This made shaping the new plate convenient. Second, the traditional steel plate was usually fixed only using a steel wire; however, the new plate could also be fixed using screws because of the arrangement of four screw holes, which can be used to fix the steel plate with the help of screws and a locking piece.

Plate accessories such as the locking piece were also improved in the modified procedure. The new locking piece was designed with three holes, two of which at each end were used for wire fixing. Generally, four wires were fixed on each side of the thorax to ensure that the locking piece was firmly fixed. The middle hole of locking piece was used to fix the locking piece with the steel plate using a screw. After the steel plate was shaped and placed through the tunnel, the surgeons could press the patient's chest wall, keeping the locking piece stable. The pressure of the steel plate and the height of the sternum could be adjusted with the help of the combination of different holes on the steel bar. This design was different from the previous locking piece designs and the traditional anti-Nuss procedure, and made the placement and fixation of the plate more convenient.

Because of the abovementioned reasons, the new steel plate was easier and more convenient to shape, implant, and fix. In our study, the time required for plate implantation and plate removal in the new procedure group was significantly shorter than that required in the traditional procedure group. The shortened operation duration may be an important factor for rapid postoperative recovery. The hospitalization duration in the new procedure group was significantly shorter than that in the traditional procedure group. The surgical procedure with the new and improved steel plate still followed the basic principles of bilateral incision, minimally invasive plate implantation, and sternal uplift. This may be an important reason for no statistically significant difference between the two groups with regard to the length of incision, cost, postoperative Haller index, and postoperative surgical outcome.

Precious studies have shown a tunnel outside the rib beneath the anterior rib muscle (2, 8, 9). This tunnel is often traumatic and causes pneumothorax (5). In our study, a subcutaneous tunnel which was outside the anterior rib muscle and beneath subcutaneous tissue was established. Two long round pliers were used from two lateral incisions to separate the subcutaneous tissue. Because the new steel plate has a shallow arc shape, it is more convenient for us to perform the procedure and less traumatic for patients to undergo the procedure.

The selection of suitable patients was also important for successful surgical correction. Before operation, we routinely performed the chest wall plasticity test wherein we let the patient stand against a wall, pressed the palm of the patient to the highest point of the breast, and observe the thoracic shape of the patient to estimate the thoracic shape of the patient after the operation. Age selection was also an important factor. We generally selected patients aged over 10 years, because patients under the age of 10 may achieve better results with external chest stents, and there was a risk of recurrence in premature surgery. The upper limit with regard to age was not fixed, and mainly depended on the patient's thoracic plasticity.

The deformable design of the steel plate made it convenient for us to shape, place, and extract the steel plate with less trauma. Moreover, this steel plate had less limitation with regard to the growth and development of the thorax in children as the steel plate would deform with the growth and development of the chest. Therefore, the shape of the chest will be better and will be maintained for a long time. This study had the limitation of a small number of patients included in the study and the study period was short. Accordingly, further high-level clinical evidence is required to evaluate the long-term applicability and benefits of using the modified anti-Nuss procedure.

In conclusion, our data show that the modified procedure for pectus carinatum has a shorter operation duration, postoperative hospitalization duration, and plate removal surgery duration. Thus, the use of the modified procedure appears to be efficient and safe as it showed no increase in the incidence of complications.

## Data Availability Statement

The raw data supporting the conclusions of this article will be made available by the authors, without undue reservation.

## Ethics Statement

The studies involving human participants were reviewed and approved by the Institutional Review Board of Xinhua Hospital (Approval No. XHEC-D-2020-106), Shanghai Jiao Tong University School of Medicine. Written informed consent to participate in this study was provided by the participants' legal guardian/next of kin. Written informed consent was obtained from the individual(s), and minor(s)' legal guardian/next of kin, for the publication of any potentially identifiable images or data included in this article.

## Author Contributions

FH and HX contributed to conception and design of the study. LW, JL, and SS contributed to data collection, analysis, and interpretation. All authors contributed in writing the manuscript and approved the final version to be published.

## Conflict of Interest

The authors declare that the research was conducted in the absence of any commercial or financial relationships that could be construed as a potential conflict of interest.

## Supplementary Material

Supplementary Figure 1 **(A)** The connecting part contains a longitudinal arrangement of four screw holes. **(B)** The steel plate can be fixed by wires as well as the screws and a locking piece. **(C)** The screw is placed next to the rib but not lodged in the rib.

Supplementary Video Procedure of the modified anti-Nuss operation using flexible plate for correcting pectus carinatum. A 15 years old male patient with pectus carinatum received modified anti-Nuss operation using flexible plate in 2019. The patient was supine and the upper limbs were abducted 90° after combined general anesthesia, tracheal intubation. We marked the highest point of the thoracic and the surgical incision on both sides. The vertical incision along the midaxillary line was adopted and the length was about 3 cm. Press the highest point of the sternum to make the profile of the chest wall as normal. Meanwhile, we measure the length between two sides of the incision, whose length was the same as the steel plate we used in the operation. After that surgical incisions on both sides along the marked line were performed and the subcutaneous tissue was separated to explore the surface of the muscle. Then we built a subcutaneous tunnel with the help of a oval forceps tied by a long rope which is reserved through the subcutaneous tunnel. Upper and lower ribs of each surgical incision are sutured with two steel wires (four wires on each side). One of the steel wire of each side is placed in the hole of the locking piece for later use. The selected steel plate was then introduced by the rope through the subcutaneous tunnel and then the rope was removed. One operator press the highest point of the sternum again, making the chest wall to the ideal state. At the same time, the other operator shaped the steel plate appropriately and place the fixing screw after aligning the fixing piece against the joint of steel plate with the screw hole at one side of the chest wall. The fixing piece was also fastened to the adjacent two ribs with steel wires The same method was performed to fix the contralateral locking piece to complete the pectus carinatum orthosis. If the thorax shape is not satisfactory, the degree of sedimentation of the chest wall could be adjusted through the lateral hole of the plate to achieve the best corrected shape. Finally, the incision was sutured and the operation complete.
