# Supplementary figures and images for: Comparison of Outcomes Between Anti-Nuss Operation and Modified Anti-Nuss Operation Using a Flexible Plate for Correcting Pectus Carinatum: A Retrospective Study
Source: Front Surg. 2021 Feb 15;7:600755. doi: 10.3389/fsurg.2020.600755 (PMC7920095; doi:10.3389/fsurg.2020.600755)

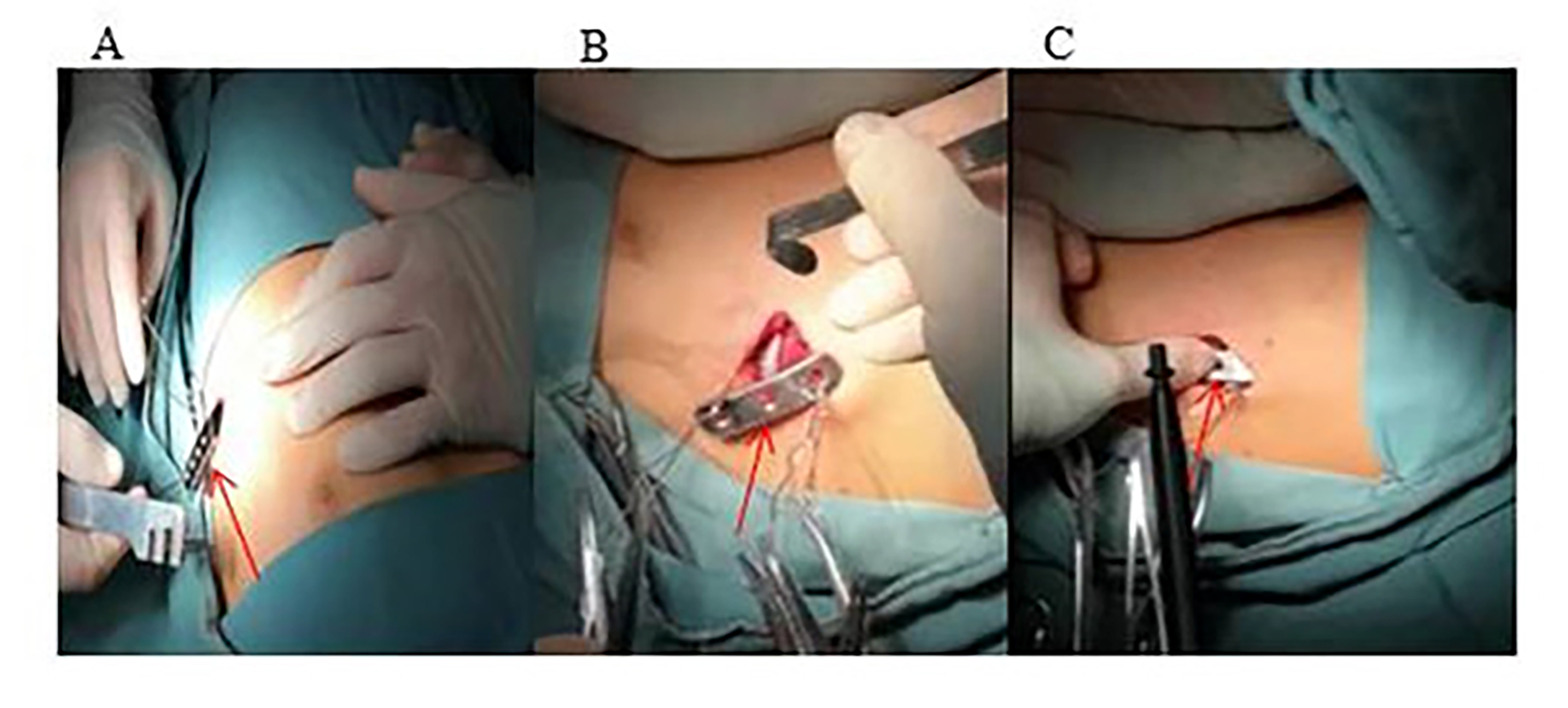

Supplement: Supplementary Figure 1 — (A) The connecting part contains a longitudinal arrangement of four screw holes. (B) The steel plate can be fixed by wires as well as the screws and a locking piece. (C) The screw is placed next to the rib but not lodged in the rib. [file Image_1.jpg]
